# Public Health Interventions for COVID-19 Reduce Kawasaki Disease in Taiwan

**DOI:** 10.3390/children8080623

**Published:** 2021-07-23

**Authors:** Ya-Ling Yang, Ho-Chang Kuo

**Affiliations:** 1 Department of Anesthesiology, Kaohsiung Chang Gung Memorial Hospital and Chang Gung University College of Medicine, Kaohsiung 83301, Taiwan; inr453@cgmh.org.tw; 2 Department of Pediatrics, Kaohsiung Chang Gung Memorial Hospital and Chang Gung University College of Medicine, Kaohsiung 83301, Taiwan; 3 Kawasaki Disease Center, Kaohsiung Chang Gung Memorial Hospital, Kaohsiung 83301, Taiwan; 4 College of Medicine, Chang Gung University, Taoyuan 33302, Taiwan

**Keywords:** public health interventions, COVID-19, SARS-CoV-2, Kawasaki disease

## Abstract

Background: Kawasaki disease (KD) is a syndrome of unknown cause that results in high fever and coronary vasculitis in children. The incidence of KD increased in Taiwan over the past few decades. Taiwanese government executed domains of early screening, effective methods for isolation or quarantine, and digital technologies for identifying potential cases for the early elimination strategy for coronavirus disease 2019 (COVID-19) and public health interventions for the severe acute respiratory syndrome coronavirus 2 (SARS-CoV-2) or COVID-19 pandemic, leading to an effective reduction of the risk of airway infections in children. The purpose of this study is to analyze whether those public health interventions reduce the incidence of KD in 2020. Methods: Patients with KD who visited Chang Gung Memorial Hospital (CGMH) between 1 January, 2018, and 31 December, 2020 were included for trend analysis. This is a retrospective case series study conducted at the CGMH, which consists of a network of seven hospital branches equipped with more than 10,000 beds in different areas of Taiwan. Results: Compared with the 2018 and 2019 databases, the incidence of KD decreased significantly by 30% and 31%, respectively (*p* < 0.05) in 2020, when public health interventions were comprehensively implemented in Taiwan. This result shows that the incidence of KD decreased during the COVID-19 pandemic in Taiwan without change of the presentation KD (typical or incomplete) and percentage of IVIG resistance in 2020. Conclusion: As public health interventions were carried out for the SARS-CoV-2 pandemic, the incidence of KD was significantly reduced in Taiwan. Is KD a preventable disease?

## 1. Introduction

Kawasaki disease (KD) is an acute febrile multisystem vasculitis that mainly affects genetically susceptible children under five years of age. Although the exact cause of KD is unclear, it is tentatively defined as an infection-immunogenetic pathogenesis [1]. The global prevalence of KD among children is the highest in Japan (218/10^5^) and the lowest (4.7/10^5^) in kids of European descent, while Taiwan’s incidence rate is 66/10^5^ [1]. During our recent study period, Taiwan’s incidence of KD more than doubled (an increase from 28.58 to 60.08 per 100,000 people) [2].

Rowley et al. have hypothesized that KD pathogenesis involves an RNA virus that usually causes asymptomatic infection but KD in a subset of genetically predisposed children [3]. Moreover, the age distribution of KD patients is highest among children under two years old and lower in those under six months old and over five years old, which is in line with the general concept of transplacental immunity and increasing immunity with age [3]. Our previous studies have also shown that the inflammatory response of microorganisms may trigger KD through toll-like receptors (TLRs) that stimulate immune-pathogenesis [4].

According to reports, the novel coronavirus is defined as severe acute respiratory syndrome coronavirus 2 (SARS-CoV-2) and has been named COVID-19 (coronavirus disease 2019) [5]. A large cross-sectional study that has been conducted on children tested for SARS-CoV-2 shows a positive rate of 3.2% [6], while more and more evidence has highlighted that the clinical severity of SARS-CoV-2 in children and young adults appears significantly milder when compared to older people with comorbidities [7,8]. However, it is reported that the number of KD cases in the United States [9], Italy [10], and the United Kingdom [11] has increased by nearly 6–10 times when compared with previous years and temporal association with SARS-CoV-2 infection [12].

SARS-CoV-2 timeline reveals that Taiwan had its first confirmed case on 21 January and the first death on 16 February of 2020 [13], and since 6 February Taiwan has started to implement public health interventions (such as keeping social distance, wearing face masks, and washing hands) among its entire population and has so far contained the SARS-CoV-2 pandemic very well. As a result, Taiwan has been highly praised for its success in protecting the interests of its citizens and controlling the pandemic [14,15]. Taiwanese government advised people to remain in Taiwan, avoid travel and domains of early screening, effective methods for isolation or quarantine, and digital technologies for identifying potential cases for the early elimination strategy for COVID-19 since February 2020. [16] Totally, there are 322 (January–March), 125 (April–June), 70 (July–September), 282 (October–December) and 799 confirmed COVID-19 cases in the year of 2020 (https://www.cdc.gov.tw/En). Fortunately, we did not have lockdown or school closure in 2020. It is also worth noting that these measures have suppressed not only the spread of SARS-CoV-2 but also other contagious diseases [17], especially for the pediatric population [13], in which contagious diseases accounted for 80% of the emergency department visits in the early months of 2020 [18]. In addition, a significant decrease of more than 50% in respiratory tract infection-related visits was found from February to April 2020 in the Taiwan national database [13]. Moreover, handwashing has long been considered an important and cost-effective infection-control method to prevent the spread of respiratory infections in healthcare and children [19]. Therefore, the aim of this study is to analyze whether the government’s public health interventions for the SARS-CoV-2 pandemic reduced the number of new KD cases in Taiwan during the COVID-19 pandemic in 2020.

## 2. Materials and Methods

### 2.1. Study Population

This is a retrospective case series study conducted at the Chang Gung Memorial Hospital (CGMH), which consists of a network of seven hospital branches, including two medical centers in Linkou and Kaohsiung city. With a total of 10,050 beds, the CGMH network is the largest and most comprehensive healthcare service provider in Taiwan, offering more than one-tenth (1/10) of medical services in the country. Included in this study were children diagnosed with KD (International Classification of Diseases–10 diagnostic code for KD (M30.3)) under the age of 18. They were KD patients who received intravenous immunoglobulin (IVIG) treatment in our network in Taiwan from January 2018 to December 2020 and who met the diagnosis criteria set by the American Heart Association [20]. IVIG resistance was defined as no defervescence (defined as a temperature >38 °C) 48 h after the completion of IVIG treatment [21,22]. The details of typical and incomplete KD are discussed in our previous paper [23]. This study was approved by the Institutional Review Board of the Chang Gung Medical Foundation (IRB number: 202100084B0).

### 2.2. Statistical Analysis

All data are expressed as mean ± standard error. Frequency was calculated by χ2 test. One-way analysis of variance with least significant (LSD) test was used for post-hoc testing to evaluate the quantitative data, while SPSS statistical software for Windows version 22 (IBM SPSS Statistics for Windows, Version 22.0., Armonk, NY, USA) was used for statistical analysis. A value of *p* < 0.05 was considered statistically significant.

## 3. Result

### 3.1. Reduction in Kawasaki Disease after Public Health Interventions for the COVID-19 Pandemic in 2020

**Figure 1:**
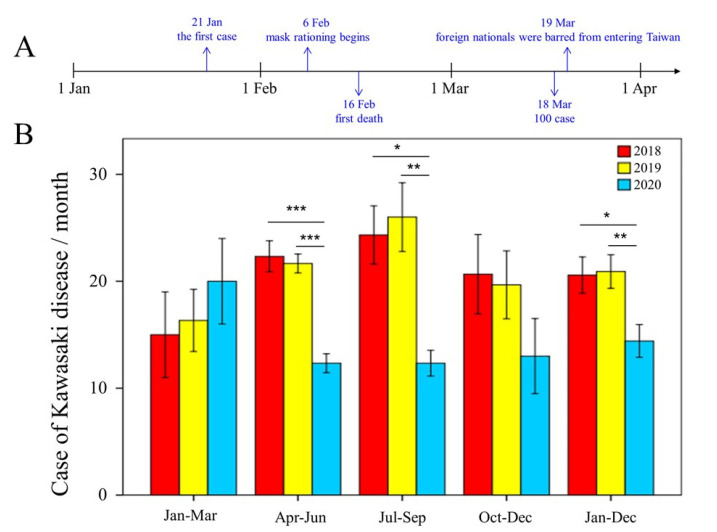
(**A**) Timeline for COVID-19 cases in Taiwan before 1 April. (**B**) Comparison of the average monthly numbers of KD cases in Chang Gung Memorial Hospital (CGMH) from 2018 to 2020. The CGMH consists of a network of seven hospital branches, with a total of 10,050 beds, and provides Taiwan’s largest and most comprehensive healthcare services. Compared with 2018 and 2019, there was no significant difference in KD cases in the first quarter of 2020 (*p* = 0.267 and 0.334, respectively), but the number of KD patients decreased significantly from April to September in 2020. All data are expressed as mean ± standard error. We used one-way analysis of variance with least significant (LSD) test for post-hoc testing to evaluate quantitative data. * Indicates *p* < 0.05, ** *p* < 0.01, *** *p* < 0.001.

This study aims to analyze whether the public health interventions for the SARS-CoV-2 pandemic reduced the number of new KD cases in Taiwan in 2020. The monthly distribution of KD cases is shown by year in Table 1, from which we observed that the total KD cases in 2020 dropped by approximately 30% and 31%, when compared to 2018 and 2019, respectively. Shown in Figure 1A is the timeline of the key SARS-CoV-2 cases and the first death in Taiwan on 16 February 2020. In addition, we have also grouped KD cases by quarter into January to March (first season), April to June (second season), July to September (third season), and October to December (fourth season), as shown in Figure 1B. During the SARS-CoV-2 pandemic, the Taiwanese government started to implement public health interventions on 6 February [14,15]. Interestingly, there is no significant difference in KD cases in the first quarter of 2020 compared to 2018 and 2019 (*p* = 0.267 and 0.334, respectively). This is evidenced by the fact that from April to September 2020, the incidence of KD decreased significantly by approximately 47.1% and 48.3%, when compared with the same period of 2018 and 2019, respectively.

### 3.2. No Change of the Presentation Kawasaki Disease and Percentage of IVIG Resistance in 2020

We further analyzed the demographic data of 175 KD (26.8% of CGMH network) patients of our KD center in the Kaohsiung-CGMH. As shown in Table 2, there was no significant difference in age, sex, KD presentation (typical or incomplete), and the percentage of IVIG resistance in 2020 compared to 2019 and 2018. (*p* > 0.05)

## 4. Discussion

In this study, we have first observed that the Chang Gung Memorial Hospital (CGMH), with its seven hospital branches in different cities in Taiwan, saw a 30% decline in KD cases in 2020, compared to 2018 and 2019. Considering the time lag effect of public health interventions in 2020, we have further observed that the number of KD cases in Taiwan from April to September 2020 dropped by 47.1% and 48.3%, compared to the same period in 2018 and 2019, respectively. Furthermore, we do not find the change of the presentation KD, including age and sex, as well as the percentages of typical KD and IVIG resistance in 2020 compared to 2019 and 2018. This may reflect that citizens were only getting started to be more vigilant and more willing to comply with the regulations of the Central Epidemic Command Center [14]. In 2020, Taiwan and Korea took similar prevention measures for COVID-19 pandemic except that the Korea government implemented a short period of school closure from February to May. Very recently, a nationwide observational study in Korea by Kang et al. revealed a reduction of about 40% in KD cases after nonpharmaceutical interventions (NPI) in the COVID-19 pandemic [24]. Nevertheless, there was no significant change in the incidence of IVIG-resistant KD during the NPI period compared with the annual past 10 years. Importantly, we also observed a drop of 44% in the number of KD cases in Taiwan from April to December 2020, compared to the same period in 2018 and 2019. These results indicate that the decrease in the incidence of KD may be attributable to the effects of reduction of infectious disease by public health interventions. However, we still cannot exclude the possibility of missed cases from patients avoiding hospitals due to a fear of COVID-19 infection. On the other hand, genetic, ethnic, and environmental factors are important in the pathogenesis of KD.

COVID-19 or SARS-CoV-2 erupted as a rapidly developing situation in China in late 2019 and has since evolved to become a worldwide pandemic. Meanwhile, cases of Kawasaki-like disease (multisystem inflammatory syndrome in children, MIS-C) have been reported to increase by nearly 6 to 10 times in New York, certain regions of Italy, and the United Kingdom, when compared with the prevalence of KD in previous years. Before the SARS-CoV-2 era, there was no evidence of a large-scale outbreak nor human-to-human transmission of KD [25]. According to reports, COVID-19 may induce MIS-C, a novel syndrome associated with SARS-CoV-2 in children [9,26,27]. Verdoni et al. reported that 8 out of 10 KD patients were tested positive for SARS-CoV-2, and the incidence of cardiac involvement was high (6/10). Coronary artery disease or aneurysm formation is the most common complication of KD, but it is rare in other children with high fever diseases. It was found that the incidence of MIS-C has increased in the United States and Europe but not in Taiwan. Before the SARS-CoV-2 pandemic, the coronavirus had been isolated in approximately 7% of KD patients [28,29]. Moreover, children with MIS-C syndrome of SARS-CoV-2 have common skin and mucosal features with KD, including strawberry tongue and edema/erythema at the extremities of the body and the site of BCG injection. Therefore, coronavirus or SARS-CoV-2 may be the cause of KD or systemic hyperinflammatory-related syndromes in patients with certain environmental and genetic backgrounds [30]. Fortunately, MIS-C cases have not been reported so far in Taiwan. A series of epidemiological explorations has suggested a negative association between the national bacillus Calmette–Guérin (BCG) vaccination policy and the prevalence and mortality of COVID-19 [31]. In Taiwan, BCG vaccination was routine performed for each baby since 1965. There were also few MIS-C reported from BCG routine vaccination countries, including Japan [32] and Korea [33]. BCG vaccination may reduce the impact of COVID-19 or MIS-C in children [34].

Is KD an infectious disease? So far, there is no answer to this question. Although KD was first reported more than 50 years ago in 1967, it is still impossible to determine the infectious agent that has clearly caused KD. There are several pieces of evidence supporting the fact that KD is a self-limited infectious disease characterized by seasonal variability and spatiotemporal clustering [1]. According to reports, various bacteria and viruses are potential causes of KD [29,35], but no consistent results have been found in different regions. In theory, the inflammatory body induced by KD can sense receptors and sensors of the innate immune system in response to external infectious microorganisms or host protein molecules [36]. In fact, researchers strongly suggest that KD is a spontaneous inflammatory-like disease with innate immune system disorders, characterized by systemic inflammation usually caused by inflammasomes [36,37,38]. Moreover, our research shows that microbial inflammatory response can trigger TLRs and stimulate immune pathogenesis in KD [4]. To fight against the SARS-CoV-2 pandemic, our government has been fully executing public health interventions, including handwashing, mask-wearing, and physical distancing. As a result, the downtrend of respiratory infections has been reflected in a statistically significant way in Taiwan [13,39]. In addition, handwashing has long been considered a vital and cost-effective infection control measure to contain the spread of enterovirus [40] and other respiratory infections in health care and community settings [19]. Our most important research result reveals that the decline in the number of KD cases is related to our government policies of wearing masks, washing hands, and keeping social distancing that has led to preventing the spread of respiratory infectious diseases in children. As SARS-CoV-2 has caused many direct deaths worldwide and still remains a concern [25], it may have caused the delay in cancer treatment due to “COVID-19 phobia”, a fear of contracting the COVID-19 virus, which keeps patients away from visiting the hospitals. Harahsheh and Burns et al. have expressed concern over the missed diagnosis or delayed diagnosis of KD during the SARS-CoV-2 pandemic [41].

Fortunately, the impact of this pandemic on Taiwan is much smaller, and all of our sick children are free to see a doctor. There is no doubt that all health workers know that wearing masks and washing hands are effective in preventing contagious diseases. In this study, we are the first to report that the reduction in person-to-person contact and the nationwide mask-wearing due to the SARS-CoV-2 pandemic have significantly reduced new KD cases by about 30% to 48%. Is KD preventable? Further research is needed to reach a conclusion.

## Figures and Tables

**Table 1:** Number of patients with Kawasaki disease.

	2018	2019	2020
January	11	11	16
February	11	17	28
March	23	21	16
April	25	23	14
May	22	22	11
June	20	20	12
July	19	32	14
August	26	25	13
September	28	21	10
October	28	16	10
November	18	26	9
December	16	17	20
Total	247	251	173

**Table 2:** Baseline characteristics of patients with Kawasaki disease.

	2018	2019	2020	*p*-Value
Cases in KD Center (% of CGMH Network)	54 (21.9)	49 (19.5)	25 (14.5)	
Male Gender (%)	53.7	51.0	64.0	0.559
Age (Year) (Mean ± SE)	2.2 ± 0.2	1.9 ± 0.3	2.2 ± 0.3	0.757
Typical KD (%)	73.1	74.5	80.0	0.801
IVIG Resistance (%)	14.8	6.1	8.0	0.319

Chang Gung Memorial Hospital (CGMH), Kawasaki Disease (KD).

## Data Availability

The datasets generated and analyzed during the current study are not publicly available due to strict ethical regulation of information privacy, but are available from the corresponding author Ho-Chang Kuo on reasonable request.

## References

[1] Wang C.L., Wu Y.T., Liu C.A., Kuo H.C., Yang K.D. (2005). Kawasaki disease: Infection, immunity and genetics. Pediatr. Infect. Dis. J..

[2] Huang Y.H., Lin K.M., Ho S.C., Yan J.H., Lo M.H., Kuo H.C. (2019). Increased Incidence of Kawasaki Disease in Taiwan in Recent Years: A 15 Years Nationwide Population-Based Cohort Study. Front. Pediatr..

[3] Rowley A.H., Shulman S.T. (2018). The Epidemiology and Pathogenesis of Kawasaki Disease. Front. Pediatr..

[4] Huang Y.H., Li S.C., Huang L.H., Chen P.C., Lin Y.Y., Lin C.C., Kuo H.C. (2017). Identifying genetic hypomethylation and upregulation of Toll-like receptors in Kawasaki disease. Oncotarget.

[5] Zhu N., Zhang D., Wang W., Li X., Yang B., Song J., Zhao X., Huang B., Shi W., Lu R. (2020). A Novel Coronavirus from Patients with Pneumonia in China, 2019. N. Engl. J. Med..

[6] Peaper D.R., Murdzek C., Oliveira C.R., Murray T.S. (2020). Severe Acute Respiratory Syndrome Coronavirus 2 Testing in Children in a Large Regional US Health System During the Coronavirus Disease 2019 Pandemic. Pediatr. Infect. Dis. J..

[7] Balasubramanian S., Rao N.M., Goenka A., Roderick M., Ramanan A.V. (2020). Coronavirus Disease (COVID-19) in Children—What We Know So Far and What We Do Not?. Indian Pediatr..

[8] Dong Y., Wang L., Burgner D.P., Miller J.E., Song Y., Ren X., Li Z., Xing Y., Ma J., Sawyer S.M. (2020). Infectious diseases in children and adolescents in China: Analysis of national surveillance data from 2008 to 2017. BMJ.

[9] Feldstein L.R., Rose E.B., Horwitz S.M., Collins J.P., Newhams M.M., Son M.B.F., Newburger J.W., Kleinman L.C., Heidemann S.M., Martin A.A. (2020). Multisystem Inflammatory Syndrome in U.S. Children and Adolescents. N. Engl. J. Med..

[10] Fabi M., Filice E., Andreozzi L., Conti F., Gabrielli L., Balducci A., Vergine G., Cicero C., Iughetti L., Guerzoni M.E. (2020). Spectrum of cardiovascular diseases in children during high peak COVID-19 period infection in Northern Italy: Is there a link?. J. Pediatr. Infect. Dis. Soc..

[11] Swann O.V., Holden K.A., Turtle L., Pollock L., Fairfield C.J., Drake T.M., Seth S., Egan C., Hardwick H.E., Halpin S. (2020). Clinical characteristics of children and young people admitted to hospital with covid-19 in United Kingdom: Prospective multicentre observational cohort study. BMJ.

[12] Consiglio C.R., Cotugno N., Sardh F., Pou C., Amodio D., Rodriguez L., Tan Z., Zicari S., Ruggiero A., Pascucci G.R. (2020). The Immunology of Multisystem Inflammatory Syndrome in Children with COVID-19. Cell.

[13] Lin C.F., Huang Y.H., Cheng C.Y., Wu K.H., Tang K.S., Chiu I.M. (2020). Public Health Interventions for the COVID-19 Pandemic Reduce Respiratory Tract Infection-Related Visits at Pediatric Emergency Departments in Taiwan. Front. Public Health.

[14] Wang C.J., Ng C.Y., Brook R.H. (2020). Response to COVID-19 in Taiwan: Big Data Analytics, New Technology, and Proactive Testing. JAMA.

[15] Lien W.C., Wu J.L., Tseng W.P., Ko P.C.-I., Chen S.Y., Tsai M.S., Chang W.T., Huang C.H., Chen S.C. (2020). Fight COVID-19 Beyond the Borders: Emergency Department Patient Diversion in Taiwan. Ann. Emerg. Med..

[16] Summers J., Cheng H.Y., Lin H.H., Barnard L.T., Kvalsvig A., Wilson N., Baker M.G. (2020). Potential lessons from the Taiwan and New Zealand health responses to the COVID-19 pandemic. Lancet Reg. Health West. Pac..

[17] Chan J.F., Yuan S., Zhang A.J., Poon V.K., Chan C.C., Lee A.C., Fan Z., Li C., Liang R., Cao J. (2020). Surgical mask partition reduces the risk of non-contact transmission in a golden Syrian hamster model for Coronavirus Disease 2019 (COVID-19). Clin. Infect. Dis..

[18] Hasegawa K., Tsugawa Y., Cohen A., Camargo C.A. (2015). Infectious Disease-related Emergency Department Visits Among Children in the US. Pediatr. Infect. Dis. J..

[19] Fung I.C., Cairncross S. (2006). Effectiveness of handwashing in preventing SARS: A review. Trop. Med. Int. Health.

[20] McCrindle B.W., Rowley A.H., Newburger J.W., Burns J.C., Bolger A.F., Gewitz M., Baker A.L., Jackson M.A., Takahashi M., Shah P.B. (2017). Diagnosis, Treatment, and Long-Term Management of Kawasaki Disease: A Scientific Statement for Health Professionals From the American Heart Association. Circulation.

[21] Huang Y.H., Lo M.H., Cai X.Y., Liu S.F., Kuo H.C. (2019). Increase expression of CD177 in Kawasaki disease. Pediatr. Rheumatol. Online J..

[22] Chang L.S., Kuo H.C. (2020). The role of corticosteroids in the treatment of Kawasaki disease. Expert Rev. Anti-Infect. Ther..

[23] Tsai C.M., Chu C.H., Liu X., Weng K.P., Liu S.F., Huang Y.H., Kuo H.C. (2021). A novel score system of blood tests for differentiating Kawasaki disease from febrile children. PLoS ONE.

[24] Kang J.M., Kim Y.E., Huh K., Hong J., Kim D.W., Kim M.Y., Jung S.Y., Kim J.H., Jung J., Ahn J.G. (2021). Reduction in Kawasaki Disease After Nonpharmaceutical Interventions in the COVID-19 Era: A Nationwide Observational Study in Korea. Circulation.

[25] Hartman H.E., Sun Y., Devasia T.P., Chase E.C., Jairath N.K., Dess R.T., Jackson W.C., Morris E., Li P., Hochstedler K.A. (2020). Integrated Survival Estimates for Cancer Treatment Delay Among Adults With Cancer During the COVID-19 Pandemic. JAMA Oncol..

[26] Kuo H.C. (2020). Kawasaki-like disease among Italian children in the COVID-19 era. J. Pediatr..

[27] Dufort E.M., Koumans E.H., Chow E.J., Rosenthal E.M., Muse A., Rowlands J., Barranco M.A., Maxted A.M., Rosenberg E.S., Easton D. (2020). Multisystem Inflammatory Syndrome in Children in New York State. N. Engl. J. Med..

[28] Dominguez S.R., Anderson M.S., Glode M.P., Robinson C.C., Holmes K.V. (2006). Blinded case-control study of the relationship between human coronavirus NL63 and Kawasaki syndrome. J. Infect. Dis..

[29] Chang L.Y., Lu C.Y., Shao P.L., Lee P.I., Lin M.T., Fan T.Y., Cheng A.L., Lee W.L., Hu J.J., Yeh S.J. (2014). Viral infections associated with Kawasaki disease. J. Formos. Med. Assoc..

[30] Berardicurti O., Conforti A., Ruscitti P., Cipriani P., Giacomelli R. (2020). The wide spectrum of Kawasaki-like disease associated with SARS-CoV-2 infection. Expert Rev. Clin. Immunol..

[31] Escobar L.E., Molina-Cruz A., Barillas-Mury C. (2020). BCG vaccine protection from severe coronavirus disease 2019 (COVID-19). Proc. Natl. Acad. Sci. USA.

[32] Fukuda S., Kaneta M., Miyake M., Ohya T., Miyakawa K., Iwamoto M., Ito S. (2021). A case of multisystem inflammatory syndrome in children in a japanese boy: With discussion of cytokine profile. Mod. Rheumatol. Case Rep..

[33] Choe Y.J., Choi E.H., Choi J.W., Eun B.W., Eun L.Y., Kim Y.J., Kim Y.H., Kim Y.A., Kim Y.K., Kwak J.H. (2021). Surveillance of COVID-19-Associated Multisystem Inflammatory Syndrome in Children, South Korea. Emerg. Infect. Dis..

[34] Curtis N., Sparrow A., Ghebreyesus T.A., Netea M.G. (2020). Considering BCG vaccination to reduce the impact of COVID-19. Lancet.

[35] Khan I., Li X.A., Law B., In U K., Pan B.Q., Lei C., Hsiao W.W. (2020). Correlation of gut microbial compositions to the development of Kawasaki disease vasculitis in children. Future Microbiol..

[36] Guo H., Callaway J.B., Ting J.P. (2015). Inflammasomes: Mechanism of action, role in disease, and therapeutics. Nat. Med..

[37] Guo M.M., Tseng W.N., Ko C.H., Pan H.M., Hsieh K.S., Kuo H.C. (2015). Th17- and Treg-related cytokine and mRNA expression are associated with acute and resolving Kawasaki disease. Allergy.

[38] Jacob C.O. (2020). On the genetics and immunopathogenesis of COVID-19. Clin. Immunol..

[39] Lee H., Lee H., Song K.H., Kim E.S., Park J.S., Jung J., Ahn S., Jeong E.K., Park H., Kim H.B. (2020). Impact of Public Health Interventions on Seasonal Influenza Activity During the SARS-CoV-2 Outbreak in Korea. Clin. Infect. Dis..

[40] Ruan F., Yang T., Ma H., Jin Y., Song S., Fontaine R.E., Zhu B.P. (2011). Risk factors for hand, foot, and mouth disease and herpangina and the preventive effect of hand-washing. Pediatrics.

[41] Harahsheh A.S., Dahdah N., Newburger J.W., Portman M.A., Piram M., Tulloh R., McCrindle B.W., de Ferranti S.D., Cimaz R., Truong D.T. (2020). Missed or delayed diagnosis of Kawasaki disease during the 2019 novel coronavirus disease (COVID-19) pandemic. J. Pediatr..

